# Characterizing Inquiries About Novel Cancer Therapies: Findings from the National Cancer Institute’s Cancer Information Service

**DOI:** 10.36401/JIPO-25-15

**Published:** 2025-10-20

**Authors:** Robin C. Vanderpool, Laura Dwyer, Diane Ng, Beth Slotman, Sandra A. Mitchell

**Affiliations:** 1 Health Communication and Informatics Research Branch, Behavioral Research Program, Division of Cancer Control and Population Sciences, National Cancer Institute, Rockville, MD, USA; 2 Cape Fox Facilities Services, Chantilly, VA, USA; 3 Public Health, Westat, Rockville, MD, USA; 4 Outcomes Research Branch, Healthcare Delivery Research Program, Division of Cancer Control and Population Sciences, National Cancer Institute, Rockville, MD, USA

**Keywords:** immunotherapy, targeted therapy, stem cell transplantation, health communication, patient education

## INTRODUCTION

Research suggests provision of timely and quality cancer information may favorably influence disease self-management, patient-centered communication, and shared decision-making between survivors, caregivers, and the oncology care team.[1,2] Cancer treatment in particular is a point at which cancer survivors, caregivers, and healthcare professionals may have significant need for information, for example, regarding available treatment options, clinical trials, and treatment side effects.[3] Information needs around treatment options are likely to be particularly acute in the current oncology landscape, where scientific advances have given rise to a range of novel regimens including immunotherapies (e.g., T-cell transfer therapy, immune checkpoint inhibitors), molecularly targeted therapies (e.g., small-molecule drugs, monoclonal antibodies, antibody-drug conjugates), and hematopoietic stem cell transplantation.[4–6] However, there has been limited study of cancer survivors’, caregivers’, and healthcare professionals’ distinct—and often unmet—information needs related to these advancements. For example, stem cell transplantation places a significant burden on caregivers, but they generally get limited support and preparation to assist them with their new responsibilities (e.g., nursing tasks, managing adverse events)[7] and may need additional information regarding what to expect postdischarge.[8] Additionally, survivors treated with immunotherapy and targeted therapies face uncertainties regarding the anticipated duration of treatment response and prognosis, and express a need for more information regarding their treatment, including logistics and possible side effects.[9–12] Healthcare professionals may also have specific information needs related to more novel or complex therapies as they discuss options with their patients or look for information on clinical trials related to these treatments.[13]

Examining information seeking about novel cancer therapies that occurs outside clinical settings could be important for better understanding and addressing the information needs of survivors, caregivers, and providers. One prominent resource for addressing questions about cancer is the National Cancer Institute’s (NCI) Cancer Information Service (CIS) (www.cancer.gov/contact). Established in 1975, the CIS is a federally supported program that provides evidence-based cancer information in English and Spanish to patients and their families, healthcare professionals, and the general public. The CIS maintains an extensive information specialist training and quality assurance program, resulting in client satisfaction scores ranging from 91–100% across all access channels and languages (Gutierrez A, personal communication, August 2025). Data collected by CIS information specialists on each client interaction can provide insights into patterns of information seeking about specific cancer topics.[13] The aim of the present analysis was to characterize the information needs expressed to the CIS by cancer survivors, caregivers, and healthcare professionals about immunotherapies, targeted therapies, and stem cell transplantation during a 6.5-year period and explore whether trends in these inquiries changed over time.

## METHODS

Cancer treatment inquiries related to immunotherapy, targeted therapy, or stem cell transplantation received by the CIS between September 2018 and February 2025 were characterized by subject(s) of interaction and client characteristics. Trends in inquiries about each of the three therapies as a percentage of all CIS treatment-related inquiries were also examined during the 6.5-year study period. Analyses were conducted in SAS 9.4 (SAS Institute, Cary, NC). Selected findings were discussed with CIS information specialists to provide qualitative contextualization of results. Because CIS programmatic data are fully deidentified, analyses were deemed nonhuman subjects research by the Westat (No. 00005551) and National Institutes of Health (No. 568158) institutional review board.

The CIS receives inquiries through four primary access channels: telephone, LiveHelp instant messaging, email, and social media. Using an electronic contact record form, CIS information specialists record information about each interaction, including subject(s) of interaction (e.g., clinical trials, managing cancer care, cancer treatment) and client type. Client types in the current analysis were limited to cancer survivors (any individual ever diagnosed with cancer), caregivers (a spouse, friend, or relative of an individual diagnosed with cancer), and health professionals (e.g., physicians, nurses, physician assistants, nurse practitioners, social workers). Additional variables of interest were language of the interaction (i.e., English, Spanish), access channel (e.g., telephone, LiveHelp), phase of the cancer control continuum (e.g., staging and treatment, posttreatment), concurrent subject(s) of the interaction other than the novel therapies of interest (e.g., side effects), cancer site(s) discussed during the inquiry (e.g., breast, lung), and referral(s) given by the CIS information specialist to the client as a result of the inquiry (e.g., to a healthcare professional to further discuss treatment options or clinical trial search results, to an organization to help with psychosocial support or financial assistance).

## RESULTS

**Figure 1:**
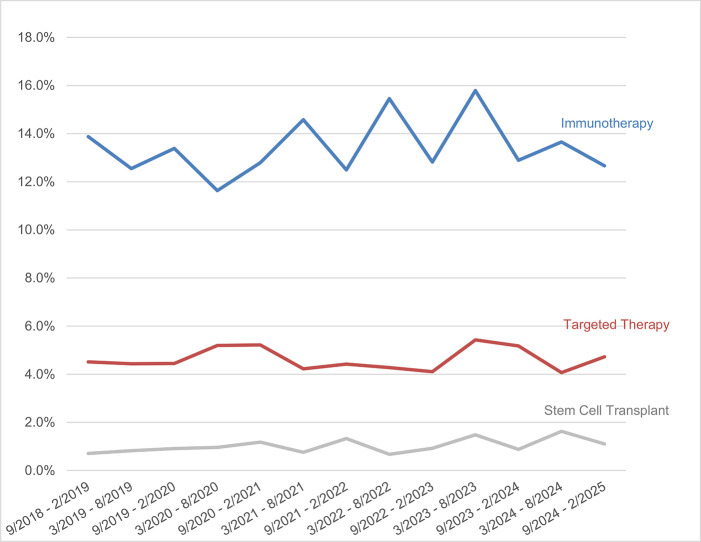
Proportion of inquiries about novel cancer therapies out of all Cancer Information Service (CIS) cancer treatment–focused inquiries, semiannual. *Note*: Subject of interaction categories are not mutually exclusive; an inquiry to the CIS may focus on more than one cancer treatment.

Between September 2018 and February 2025, the CIS received a total of 23,500 cancer treatment–related inquiries from survivors, caregivers, and health professionals. Specifically, 3146 inquiries were related to immunotherapy (13.4%); 1080 were about targeted therapy (4.6%); and 228 were about stem cell transplantation (0.97%). The remaining treatment-related inquiries centered on general cancer treatment questions (59.3%), chemotherapy (10.1%), radiation therapy (9.0%), surgery (6.7%), and hormonal therapy (4.3%). (*Note:* Subject of interaction categories are not mutually exclusive; an inquiry to the CIS may focus on more than one treatment, resulting in the total percentage of cancer treatment-related discussions exceeding 100%.) As illustrated in Figure 1, during the study period, inquiries about immunotherapy ranged from 11.6–15.8%, followed by targeted therapy (4.1–5.4%) and stem cell transplantation (0.7–1.6%), indicating relative stability.

As shown in Table 1, more than half of all CIS contacts about these three therapy types were from caregivers, whereas health professionals accounted for fewer than 5% of contacts. Notably, caregivers accounted for two-thirds of the stem cell transplantation inquiries (65.4%). Although most inquiries about the three therapies of interest were in English (75.9–91.7%), nearly one-quarter of inquiries about stem cell transplantation (24.1%) were from Spanish-language clients, compared to only 11.1% of immunotherapy and 8.3% of targeted therapy inquiries. Overall, telephone (48.1–51.6%) and LiveHelp (25.4–30.6%) were the CIS communication channels most frequently used to inquire about these therapies.

**Table 1:** Descriptive Findings of CIS Inquiries Involving a Discussion of Immunotherapy, Targeted Therapy, or Stem Cell Transplantation, September 2018–February 2025

	Immunotherapy		Targeted Therapy		Stem Cell Transplantation	
	*n*	%	*n*	%	*n*	%
All relevant CIS inquiries	3146	100	1080	100	228	100
Client type						
Caregiver	1823	58.0	591	54.7	149	65.4
Cancer survivor	1253	39.8	461	42.7	70	30.7
Health professional	70	2.2	28	2.6	9	4.0
Language						
English	2796	88.9	990	91.7	173	75.9
Spanish	350	11.1	90	8.3	55	24.1
Access channel						
Telephone	1512	48.1	557	51.6	116	50.8
LiveHelp	891	28.3	330	30.6	58	25.4
Email	691	22.0	188	17.4	49	21.5
Social media	52	1.7	5	0.5	5	2.2
Phase of the cancer control continuum						
General public or undiagnosed	13	0.4	*	*	*	*
Screening or diagnostic	49	1.6	11	1.0	*	*
Staging or treatment	2809	89.3	988	91.5	200	87.4
Posttreatment	104	3.3	27	2.5	13	5.7
End of life	40	1.3	14	1.3	*	*
No cancer continuum coded	131	4.2	37	3.4	9	4.0
Concurrent subjects of interaction†						
Clinical trials	1426	45.3	373	34.5	53	23.3
Managing cancer care	545	17.3	230	21.3	70	30.7
Understanding cancer or general cancer	285	9.1	119	11.0	21	9.2
Side effects	133	4.2	56	5.2	9	4.0
Cancer sites†						
Digestive or gastrointestinal	701	22.3	218	20.2	16	7.0
Breast	491	15.6	167	15.5	16	7.0
Genitourinary	438	13.9	107	9.9	11	4.8
Lung	323	10.3	131	12.1	*	*
Hematologic or blood	284	9.0	141	13.1	140	61.4
Melanoma	152	4.8	20	1.9	*	*
Referrals†						
Healthcare professional (e.g., doctor, nurse, pharmacist, social worker)	2664	84.7	916	84.8	173	75.9
National or community organization or other government program	575	18.3	252	23.3	82	36.0
International referral	524	16.7	127	11.8	42	18.4
NCI-designated cancer center	373	11.9	142	13.2	34	14.9

* *n* < 5 suppressed with secondary suppression applied where needed.

† Subject of interaction, cancer site, and referral categories are not mutually exclusive and multiple categories can be coded for each CIS inquiry. The categories under each of these variables represent those with the highest percentages in the current analysis and are not comprehensive of all categories captured by the CIS.

CIS, Cancer Information Service; NCI, National Cancer Institute.

Approximately 90% of inquiries made about these novel treatments arose in the context of the staging or treatment phase of the cancer continuum. The most common concurrent subjects of interaction included clinical trials (e.g., trial options, eligibility, enrollment), followed by managing cancer-related care (e.g., finding healthcare services, financial assistance) (Table 1). Comparatively, immunotherapy inquiries included more discussion about clinical trials (45.3%), whereas stem cell transplantation inquiries more often involved discussions about managing care (30.7%). Roughly 5% of inquiries about these three therapies focused on treatment side effects. There was also variability across the three therapies in terms of the cancer sites discussed. For example, digestive or gastrointestinal cancer was the most commonly discussed cancer in immunotherapy (22.3%) and targeted therapy (20.2%) inquiries, whereas hematologic or blood cancer comprised the majority (61.4%) of the stem cell transplantation discussions. Across the three therapies, CIS information specialists most commonly referred clients to healthcare professionals (75.9–84.8%), followed by national, community, and/or government programs (18.3–36.0%).

## DISCUSSION

This descriptive study is one of the first assessments of cancer information seeking related to immunotherapy, targeted therapy, and stem cell transplantation conducted in a large, national sample of survivors, caregivers, and health professionals. Although these novel treatments comprised a relatively small proportion of overall cancer treatment–related inquiries to the CIS—and remained stable during the 6.5-year study period—the findings highlight several important considerations. For example, immunotherapy inquiries were more prevalent than inquiries about targeted therapy and stem cell transplantation. Although the exact reason for greater interest in immunotherapy cannot be ascertained from CIS data, information specialists report that increased media coverage of this particular therapy may have influenced clients’ beliefs that immunotherapy is more “natural” because it uses the body’s own immune system to fight cancer and therefore may help patients avoid chemotherapy and radiation therapy. These perceptions may contribute to increased interest in immunotherapy among individuals contacting the CIS. Importantly, information specialists are trained to help dispel related myths and provide evidence-based education about these therapies.

Additionally, over half of CIS inquiries about these three therapies came from caregivers, indicating that they were seeking additional information and resources related to treatment options outside the clinical setting. This observation is consistent with a study that found caregivers of patients receiving immunotherapy generally wanted more information than the patients themselves.[10] Notably, caregivers accounted for most stem cell transplantation inquiries, which may reflect the significant caregiving burden of this treatment (e.g., managing medications, maintaining strict dietary requirements).[7,14] Beyond specific cancer site and treatment information, CIS information specialists are also trained to respond to both caregivers’ and survivors’ concerns about financial hardships, provide emotional support, and assist with seeking additional medical opinions, among other issues. As such, the CIS commonly provides referrals to oncology social workers, NCI-designated cancer centers, and programs such as the Caregiver Action Network, Family Caregiver Alliance, Cancer*Care*, the National Bone Marrow Transplant Link, and Be the Match.

The analysis also found that Spanish language inquiries represented nearly one-quarter of CIS discussions about stem cell transplantation. The CIS has a long history of providing language-concordant cancer information to the public and strives to meet the treatment-related informational needs of its Spanish-language clients through bilingual communication exchanges and translated online content and publications. Cancer education efforts should prioritize ensuring access to novel treatment information in different languages via multiple communication channels, for example, through the integration of artificial intelligence (AI) for language translation.[15]

A notable proportion of CIS inquiries about these novel therapies also discussed clinical trials, particularly when the inquiry was about immunotherapy or targeted therapy, suggesting there may be an unmet need for clinical trial information. Previous research has shown that clinical trials are a common CIS discussion topic, particularly among survivors and caregivers.[13] For five decades, the CIS has prioritized the provision of clinical trial information to the public; information specialists are trained to conduct tailored clinical trial searches (e.g., via clinicaltrials.gov) and offer follow-up services to determine if additional support is needed. The CIS can serve as a valuable and trusted resource to support survivors, caregivers, and healthcare professionals in shared decision-making about emerging therapies and trial participation.

Given the symptoms and adverse effects associated with the treatments of interest, it was expected that many inquiries would include a concurrent focus on symptom management and treatment side effects; however, the data did not support this assumption. Information specialists report that patients and caregivers commonly contact the CIS when they are exploring treatment options or gathering information to discuss during a future medical appointment. As such, awareness of possible side effects may be limited, and the client’s focus is instead on understanding whether these therapies might be a viable treatment option for them and how and where to access these treatments.

The limited number of inquiries from health professionals was also surprising and may signal an assumption that the CIS focuses on patients and caregivers. It may also reflect the fact that professional organizations (e.g., American Society of Clinical Oncology, National Comprehensive Cancer Network, Society for Immunotherapy of Cancer) have published resources to meet the information needs of providers regarding these novel therapies, supporting them in managing toxicities and other aspects of clinical care during and following treatment.[16]

These results should be considered in light of several limitations. We were unable to analyze nuanced aspects of CIS clients’ information needs beyond the standard categories that are recorded by information specialists to describe each interaction. Due to federal Office of Management and Budget policies to reduce survey burden on the public, collection of sociodemographic characteristics of CIS clients is also limited. Additionally, the sample is not nationally representative and likely reflects populations who have access to telephone and computer technologies. These limitations are balanced by the strengths of this data source, including the use of real-world data derived from inquiries made to a nationally available and reputable cancer information service.

## CONCLUSION

Findings from this multiyear, exploratory analysis profiling over 4450 CIS inquiries from survivors, caregivers, and health professionals about immunotherapy, targeted therapy, and stem cell transplantation demonstrate the need for trusted, readily available information about these treatments. As novel and precision cancer therapies continue to be integrated into oncology care and shared decision-making about treatment selection becomes more complex, person-centered communication and culturally appropriate information provision will remain essential. Being knowledgeable about novel cancer treatments can support patients’, caregivers’, and healthcare providers’ engagement in clinical conversations and help guide expectations about treatment and side effects. This study highlights the important role CIS plays as a resource for information about novel cancer therapies for survivors, caregivers, and providers. Efforts to further enhance the CIS, for example, through implementation of AI tools that triage client queries, provide real-time analysis of and response to voice and online chat conversations, and provide after-hours assistance, could help ensure individuals have access to needed information in the most timely and efficient manner possible. Ensuring patients and caregivers have their cancer information needs addressed—particularly in a time of rapid scientific advancement in therapeutics and information technologies—is fundamental to enhancing patient-provider relationships, strengthening shared decision-making processes, and improving cancer outcomes.

## Data Availability

Data to support this study are available upon reasonable request to the corresponding author.

## References

[1] Schulman‐Green D Jeon S Managing cancer care: a psycho‐educational intervention to improve knowledge of care options and breast cancer self‐management. *Psychooncology*. 2017;26:173–181. 26537980 10.1002/pon.4013

[2] Reyes KR Wong P Petrofsky M et al. Shared decision-making needs, barriers, and facilitators of patients with newly diagnosed advanced cancer in the hospital: a multi-level, mixed-methods study. *Support Care Cancer*. 2024;32:315. 38684522 10.1007/s00520-024-08515-1 PMC11058864

[3] Fletcher C Flight I Chapman J et al. The information needs of adult cancer survivors across the cancer continuum: a scoping review. *Patient Educ Couns*. 2017;100:383–410. 27765377 10.1016/j.pec.2016.10.008

[4] Xu S Temitope O Woo AK et al. Current activity trends and outcomes in hematopoietic cell transplantation and cellular therapy—a report from the CIBMTR. *Transplant Cell Ther*. 2025;31:505–532. 40398621 10.1016/j.jtct.2025.05.014 PMC12302970

[5] Liu B Zhou H Tan L et al. Exploring treatment options in cancer: tumor treatment strategies. *Signal Transduct Target Ther*. 2024;9:175. 39013849 10.1038/s41392-024-01856-7 PMC11252281

[6] Scott EC Baines AC Gong Y et al. Trends in the approval of cancer therapies by the FDA in the twenty-first century. *Nat Rev Drug Discov*. 2023;22:625–640. 37344568 10.1038/s41573-023-00723-4

[7] Applebaum A Sannes T Mitchell H et al. Fit for duty: lessons learned from outpatient and homebound hematopoietic cell transplantation to prepare family caregivers for home-based care. *Transplant Cell Ther*. 2023;29:143–150. 36572386 10.1016/j.jtct.2022.12.014 PMC9780643

[8] Foster J Moore H Preussler JM et al. Information needs for treatment decision-making of hematopoietic cell transplant patients 65 years or older and caregivers. *J Cancer Educ*. 2020;35:651–660. 30877651 10.1007/s13187-019-01506-5

[9] Zwanenburg LC Suijkerbuijk KPM van Dongen SI et al. Living in the twilight zone: a qualitative study on the experiences of patients with advanced cancer obtaining long-term response to immunotherapy or targeted therapy. *J Cancer Surviv*. 2024;18:750–760. 36495465 10.1007/s11764-022-01306-9 PMC11082039

[10] Boulanger MC Falade AS Hsu K et al. Patient and caregiver experience with the hope and prognostic uncertainty of immunotherapy: a qualitative study. *JCO Oncol Pract*. 2025;21:178–187. 39038253 10.1200/OP.24.00299 PMC11751125

[11] Kutzleben MV Galuska JC Hein A et al. Needs of lung cancer patients receiving immunotherapy and acceptance of digital and sensor-based scenarios for monitoring symptoms at home—a qualitative-explorative study. *Int J Environ Res Public Health*. 2022;19:9265. 35954619 10.3390/ijerph19159265 PMC9368591

[12] Petrillo LA Shimer SE Zhou AZ et al. Prognostic communication about lung cancer in the precision oncology era: a multiple-perspective qualitative study. *Cancer*. 2022;128:3120–3128. 35731234 10.1002/cncr.34369

[13] Vanderpool RC Ng D Huang G et al. Disparities in cancer clinical trials information-seeking: findings from the National Cancer Institute’s Cancer Information Service. *Patient Educ Couns*. 2024;127:108358. 38936161 10.1016/j.pec.2024.108358 PMC11323059

[14] Song Y Chen S Roseman J et al. It takes a team to make it through: the role of social support for survival and self-care after allogeneic hematopoietic stem cell transplant. *Front Psychol*. 2021;12:624906. 33868091 10.3389/fpsyg.2021.624906 PMC8044751

[15] Lion KC Lin Y-H Kim T Artificial intelligence for language translation: the equity is in the details. *JAMA*. 2024;332:1427–1428. 39264601 10.1001/jama.2024.15296

[16] Haanen J Obeid M Spain L et al. Management of toxicities from immunotherapy: ESMO Clinical Practice Guideline for diagnosis, treatment and follow-up. *Ann Oncol*. 2022;33:1217–1238. 36270461 10.1016/j.annonc.2022.10.001

